# Did new transmission cycles in anthropogenic, dense, host populations encourage the emergence and speciation of pathogenic Bordetella?

**DOI:** 10.1371/journal.ppat.1007600

**Published:** 2019-03-28

**Authors:** Kalyan K. Dewan, Eric T. Harvill

**Affiliations:** Department of Infectious Disease, College of Veterinary Medicine, University of Georgia, Athens, Georgia, United States of America; Duke University School of Medicine, UNITED STATES

Until the advent of vaccines in the mid 1950s, whooping cough (pertussis) was among the most prevalent and deadly diseases for children in the United States [1, 2] and still remains a worldwide problem, particularly for developing countries [3]. But unlike similarly distinctive human diseases described thousands of years earlier, records of whooping cough emerged only a few hundred years ago. The severe and distinctive cough facilitates such rapid spread of *Bordetella pertussis* that epidemics rapidly burn through populations and require a critical community size large enough to sustain the organism through interepidemic periods [4]. The concept that increasingly dense and interconnected human populations facilitated the emergence of the virulent form of *B*. *pertussis* can be applied to the emergence of other *Bordetella* species, as discussed below.

## The origins of *B*. *pertussis*

The closest phylogenetic neighbors of *B*. *pertussis* are *B*. *parapertussis* and *B*. *bronchiseptica*, the three species sharing genetic similarities up to 99% in large sections of the conserved genomic regions, and together commonly referred to as the Classical Bordetellae [5]. The current view of their natural history, based on phylogenetic assessment, is that the ancestral progenitor of the Classical Bordetellae was likely to be similar to the broad host range *B*. *bronchiseptica*. The human-restricted *B*. *pertussis* and *B*. *parapertussis* emerged independently from it [5,6], as did another lineage, also called *B*. *parapertussis* (Bpp_ov_) that only infects sheep [7].

The Classical Bordetellae are able to infect the respiratory tracts of mammals, sharing a seemingly simple system to regulate all the genes involved. Over three decades ago, Weiss and Falkow discovered Bvg, the prototypical two component system that mediated a transition between two profoundly different virulence “phases” in *B*. *pertussis* [8]. Quite understandably, the study of the classical *Bordetella* species has focused on the virulent (Bvg^+^) phase, in which “virulence factors” involved in interactions with mammalian hosts are expressed [9, 10]. However, a puzzle remained; hundreds of genes that are not necessary for survival in mammals were found to be induced at environmental temperatures in a “nonvirulent” (Bvg^−^) phase. Based on this observation, Weiss and Falkow speculated presciently about some environmental niche outside the mammalian host. But only recently did a search for progenitor *Bordetella* species amongst metagenomic databases reveal genetic traces of such in soil, water, protists, and plants, strongly suggesting an environmental origin of the genus [11].

## Independent, interconnected transmission cycles in ancestral Bordetellae

**Fig 1:**
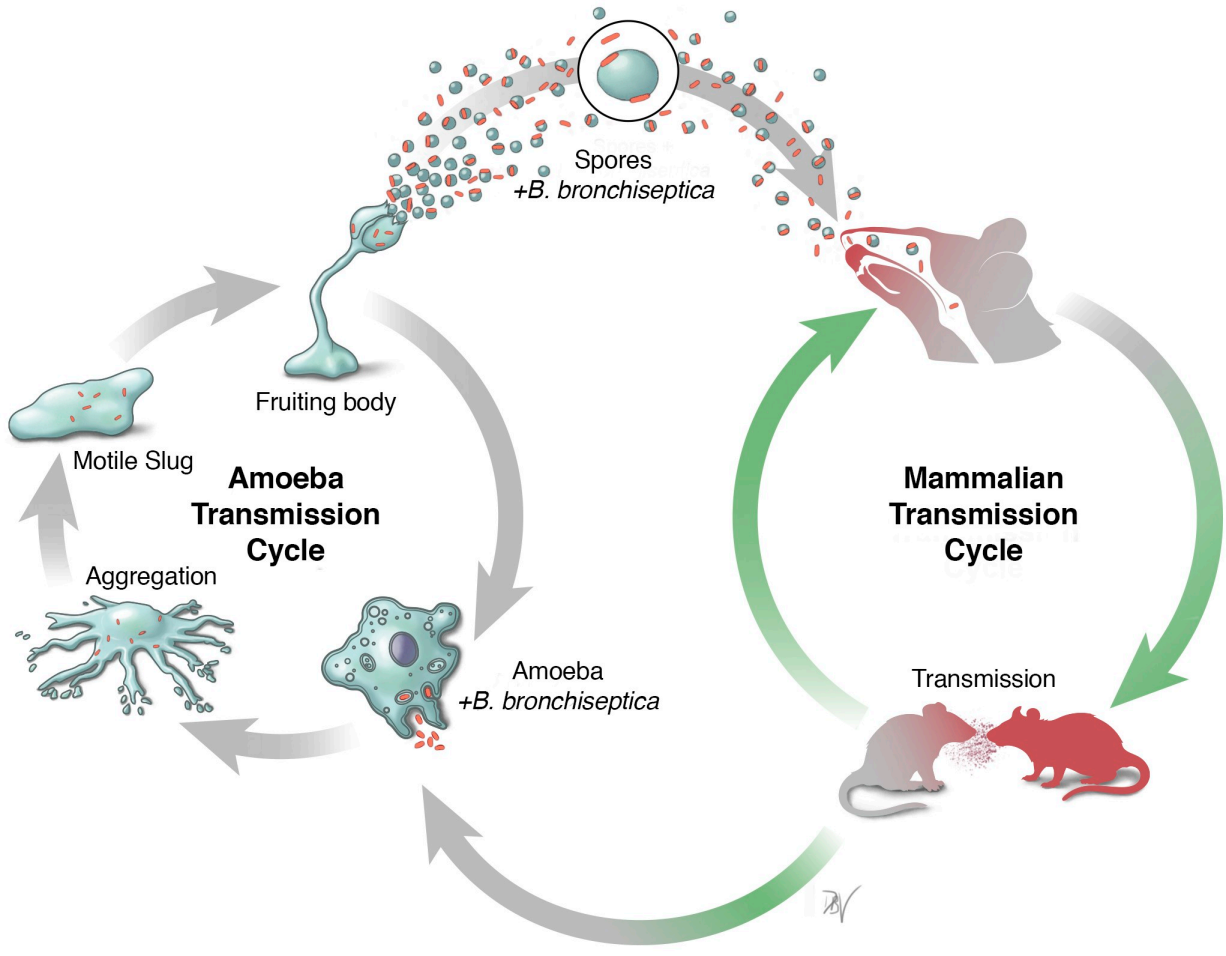
Independent but interconnected life cycles of *Bordetella bronchiseptica*. *B*. *bronchiseptica* (red rods) can infect a range of mammalian species as a respiratory pathogen. Alternately, it can associate with predatory amoeba to expand in numbers and disperse in the environment via their complex life cycle.

Evidence of likely environmental origins of the ancestral lineage of pathogenic *Bordetella* species led to a recent study that uncovered two independent and complete but intersecting transmission cycles for *B*. *bronchiseptica* (Fig 1) [12]. One cycle (Bvg^+^) involves circulating as a respiratory pathogen and/or commensal among a broad range of domesticated and wild mammals. The other life cycle (Bvg^−^) enables a stable association between the bacteria and predatory amoeba in the extra-host environment, allowing *B*. *bronchiseptica* to grow and disperse to new locations along with the amoebae. Experiments have also shown that modest numbers of amoeba and spores that harbor the bacteria can colonize mammalian hosts via drinking water, indicative of the ability of *B*. *bronchiseptica* to switch between these alternating life cycles in the wild [12]. Occasional spillover from one life cycle to the other creates an observable “meta” cycle of transmission that can explain both the Bvg paradox and some of the remarkable abilities of the various *Bordetella* species [13].

## Anthropogenic sources of dense host populations favor closed life cycles

The remarkable ability to subvert phagocytic amoebae of the environment is likely to involve many different molecular “tools” that could contribute to the ability to cause opportunistic infections in mammals. Transmission between animal hosts is facilitated by pathogenic symptoms such as cough or rhinorrhea. But a host population must be sufficiently dense and large to maintain a constant, uninterrupted chain of transmission that lasts over many generations, allowing for extended evolution, specialization, and speciation. Ancient wild animal populations were relatively sparse with less opportunity for sustained transmission chains, so a closed life cycle only in animals was doomed for extinction, hence the “meta” cycle was maintained. But relatively recent increases in density and size of various animal populations, a profound effect of the rise of the Anthropocene, would predictably affect the opportunity for emergence of pathogenic *Bordetella* species with closed life cycles. In this light, the emergence and/or expansion of *Bordetella* species as pathogens of individual host animals may be viewed as the consequence of human activities such as animal herding, poultry farming, and urbanization.

## Host restriction and speciation—A recurring theme for Bordetellae species

**Fig 2:**
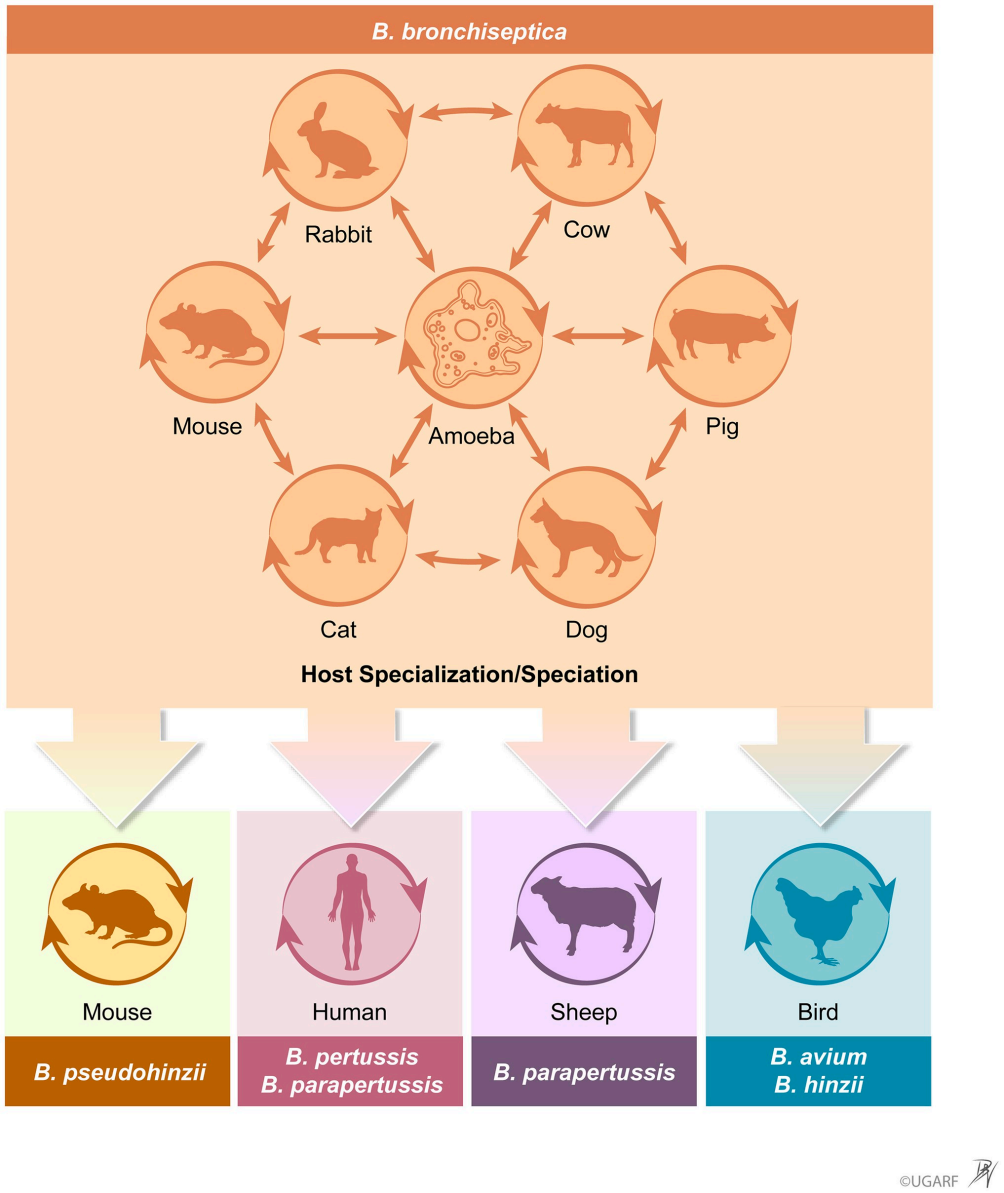
A *Bordetella* metacycle of transmission: The source of host-restricted species. Schematic of a progenitor *Bordetella* species involved in a complex metacycle of transmission involving alternative cycles in environmental protists or animal host species. Phylogenetic relations strongly indicate that host specializations of transmission restricted species, including *B*. *pertussis*, evolved from a metacycle of transmission of a *B*. *bronchiseptica*-like ancestor. [Image by: Danielle Brittany Vanbrabrant ©2019—University of Georgia Research Foundation, Inc.].

Repeated speciation correlating with host restriction is observed in several instances amongst both the Classical and non–Classical Bordetellae, as well as in a wide range of different animal hosts (Fig 2). In addition to *B*. *pertussis* and *B*. *parapertussis*_*Hu*_ found transmitting only among humans and *B*. *parapertussis*_*Ov*_ only among sheep, the nonclassical species *B*. *avium* and *B*. *hinzii* have been found naturally circulating among birds, whereas the newly discovered species, *B*. *pseudohinzii*, has been found naturally circulating among rodents, including laboratory mice [14]. The phylogenetic relationships of these species [15] indicate that such host-specialized transmission cycles have arisen from a common ancestral metacycle in the environment.

The adaptive changes accompanying host restriction in the various *Bordetella* species has not been without genetic consequences. The genome size reduction seen in *B*. *pertussis* (approximately 23%) and *B*. *parapertusis* (approximately 11%) compared to *B*. *bronchiseptica* (strain RB50) [5] appears to be a striking result of the commitment to one closed life cycle, transmitting amongst human hosts, while abandoning at least some aspects of the other life cycle. More diverse species not committed to a closed life cycle have not undergone such genome reductions. These genetic changes among the host-restricted Bordetellae appear to involve Insertion Sequence elements that have mediated extensive genome rearrangements including the large-scale loss of genetic material [16]. Similar large genome-size reductions also appear to have occurred in the emerging human respiratory pathogen *B*. *holmesii* [15] and in *B*. *avium* [17] with its commitment to a closed life cycle in birds.

However, despite the genome reduction and the loss of its environmental existence, the master regulator of the Bvg system controlling the switch from the Bvg^+^ to the Bvg^−^ phase, and corresponding transcriptional response of Bvg^−^ phase genes in environmental temperatures remains conserved among the human restricted classical species. These observations indicate some modest role of Bvg^−^ phase genes, traditionally believed to be unnecessary in the mammalian host. It is possible that some mechanisms that enabled the ability to survive intracellularly within amoebae in soil and/or water (Bvg- phase) continues to contribute to the ability to overcome mammalian macrophage defenses, in keeping with the hypothesis that amoeba has served as the ancient training ground of modern-day pathogens [18, 19].

## Bordetellae metacycles of transmission: A reservoir of human pathogens?

The search for the origins of *B*. *pertussis* has revealed several potential progenitor *Bordetella* species in the environment [11]. Their presence in widely varying environmental niches, such as contaminated soil and surfaces of oil paintings makes it clear that non–Classical Bordetellae are not stagnant and/or vestigial remnants of prior states but vibrant and aggressively evolving organisms that are well adapted and successful in different niches. There are anecdotal reports of nonclassical *Bordetella* species being isolated from humans *(B*. *holmesii*, *B*. *hinzii*, *B*. *trematum*, *B*. *bronchialis*, *B*. *flabilis*, *B*. *sputigena*, *B*. *ansorpii*, *B*. *petrii)* [20–24]. Such clinical cases are often described as instances of opportunistic infections associated with immune-deficient states. However, given the diversity of opportunistic pathogens that have been observed, and the likelihood that there is substantial under-reporting, the true numbers or range of species that humans host can only be much larger. Based on a broader view of the natural history of *Bordetella* species, these cases could be described as spillover from various established transmission cycles in mammals or the environment. Though capable of partially overcoming mammalian host defenses using mechanisms likely acquired in the environment, most appear to lack specialized mechanisms to efficiently transmit among humans. One likely hurdle they face is the substantial current vaccine- or infection-induced immunity to *B*. *pertussis*. If it is a goal to eliminate *B*. *pertussis* from human populations [25], then it should be considered that other *Bordetella* species may be emerging from diverse sources, including potential metacycles of transmission in the environment. Anthropogenesis of increasingly high-density animal populations could affect the ongoing evolution of virulence in these sources of zoonoses.
